# Supplementary material for: The development of narrative skills in Turkish-speaking children: A complexity approach
Source: PLoS One. 2020 May 6;15(5):e0232579. doi: 10.1371/journal.pone.0232579 (PMC7202631; doi:10.1371/journal.pone.0232579)
Supplement: S1 Fig — (DOCX) [file pone.0232579.s001.docx]

**S1 Fig. Hypothesized relations between cognitive abilities (predictors) and types of complexity (outcomes) during overall development from 4-11 years of age.** The relations between predictors and outcomes may be modulated by gender.

Cognitive abilities (Predictor Tasks) Domains of complexity (outcomes)

Plot

complexity

Executive Function

(Emotional Stroop Task)

Evaluative complexity

Theory of mind

(1^st^ order, 2^nd^ order ToM)

Syntactic complexity

Linguistic recursivity

(Comprehension of complements clauses Task)

development
