# Supplementary material for: The development of narrative skills in Turkish-speaking children: A complexity approach
Source: PLoS One. 2020 May 6;15(5):e0232579. doi: 10.1371/journal.pone.0232579 (PMC7202631; doi:10.1371/journal.pone.0232579)
Supplement: S1 Table — (DOCX) [file pone.0232579.s002.docx]

**S1 Table. Coding manual for plot complexity (adapted from Ayas-Koksal, 2011, p. 38-39)**

| Main Components | Subcomponents | Points |
| --- | --- | --- |
| 1) Plot onset | Preceding event/setting | 1 |
|  | Temporal location | 1 |
|  | Introduction of 3 main characters (boy, dog, frog) | 1 - 3 |
|  | Introduction of the main event (Frog’s disappearance) | 1 |
|  | Realization of the main event by the character(s) | 1 |
|  | Reaction of the character(s) to the main event | 1 |
| 2) Plot unfolding | Searching for the frog and related adventures   - at home | 1 |
|  | - encountering animals in the forest (4 animals) | 1 - 4 |
|  | - falling down | 1 |
| 3) resolution | Finding the frog | 1 |
| 4) search theme | Explicitly stating the lost frog | 1 |
|  | Searching for the lost frog | 1 |
|  | Reiteration of search theme | 0 - 2 |
| Total points | | 19 |
