# Supplementary material for: The development of narrative skills in Turkish-speaking children: A complexity approach
Source: PLoS One. 2020 May 6;15(5):e0232579. doi: 10.1371/journal.pone.0232579 (PMC7202631; doi:10.1371/journal.pone.0232579)
Supplement: S2 Table — (DOCX) [file pone.0232579.s003.docx]

**S2 Table. Categories of evaluative devices coded for evaluative complexity.**

| Category | Definition | Examples |
| --- | --- | --- |
| 1) mental state terms | internal states of  the story characters |  |
| 1. emotional states | Emotive states | *kızmak* ‘get angry’, *mutlu* ‘happy’, *üzülmek* ‘become sad’, *korkmak* ‘be scared of’, *sevmek* ‘like’ |
| 1. affect expression | Expression of affect | *gülümsemek* ‘smile’, *sarılmak* ‘hug’ |
| 1. motivation and ability | Expression of intention, desire, and ability | *istemek* ‘want’, *çalışmak* ‘try’, *-AbIlmek* ‘be able, can’ |
| d) cognitive states | Cognitive mental states | *Düşünmek* ‘think’, *karar vermek* ‘decide’, *bilmek* ‘know’ |
| 2) hedges | Expression of uncertainty of the narrator for the content of his/her statement | *galiba* ‘probably’, *belki* ‘maybe’, *zannediyorum* ‘I assume’ |
| 3) negative qualifiers | Any direct negation indicating the discrepancy between the narrator’s expectations and what happened in the story | *Kurbağayı burada bulamadılar* ‘they could not find the frog here’ |
| 4) character speech | Direct and indirect statements of the utterances of the story characters | *Çocuk “kurbağa, neredesin?” diye seslendi* ‘the boy shouted “Frog, where are you?” |
| 5) enrichment expressions | Adverbial phrases for unexpected/inferred nature of an action | *Yine* ‘again’, intensifiers such as *çok* ‘very’, *her* ‘every’; repetitions |
| 6) evaluative remarks | Expression of the subjective point of view of the narrator | *Tabii ki de köpek sahibi ile beraber yatakta yatıyordu* ‘of course, the dog is lying together with his owner in bed’ |
| 7) causal expressions | Expression of the inferred causes of events and actions | *Çünkü* ‘because’, *için* ‘for’ |
| 8) contrastive expressions | Expression of unexpected /contrastive events | *ama*, *fakat* ‘but’ |
