# Supplementary material for: The development of narrative skills in Turkish-speaking children: A complexity approach
Source: PLoS One. 2020 May 6;15(5):e0232579. doi: 10.1371/journal.pone.0232579 (PMC7202631; doi:10.1371/journal.pone.0232579)
Supplement: S3 Table — (DOCX) [file pone.0232579.s004.docx]

| Type of clauses | Suffixes/conjunctions | Example* |
| --- | --- | --- |
| Adverbial clauses | Converbs not marked for person, formed with suffixes  -(y)IncA^[[1]](#footnote-1)^, -(y)ken,  -(y)ArAk, -Ip | köpek de kokla**yarak** kurbağayı bulmaya çalışıyordu.  ‘through sniffing, the dog tried to find the frog’ |
|  | converbs marked for person, formed with suffixes -DIK,  -(y)AcAK | Uyan**dık**larında çok şaşırıyorlar.  ‘When they woke up, they became very surprised’ |
|  | finite adverbial clauses formed with *diye* and *ki*. | Baykuş gelmesin **diye** küçük çocuk kaçmaya çalışıyordu.  ‘the little boy was trying to escape so that the owl did not come’ |
| Noun clauses | formed with suffixes  –mE, -mEk, -Iş | Kurbağa kavanozdan çıkmak istemiş.  ‘The frog wanted to escape from the jar’ |
|  | Complement clauses | Bunun kendi kurbağalarının sesi olabileceğini düşünüyorlar.  ‘they thought that it might be the voice of their own frog’ |
|  | Direct speech clauses | Çocuk “Ooh, kurbağayı bulduk” demiş.  ‘The boy said “Ohh, we found the frog”.’ |
| Relative clauses | Formed with -DIK,-AcAK, -En | O kutunun içine koy**duğ**u hayvan yok.  ‘the animal that he put into that box was missing.’ |

**S3 Table. Coding scheme for the subordinate clauses in narratives.**

*Examples were taken from the participants of the present study.

1. Affixes alternate according to the rules of vowel harmony. Alternating vowels and consonants are represented by uppercase characters. [↑](#footnote-ref-1)
