# Supplementary material for: The development of narrative skills in Turkish-speaking children: A complexity approach
Source: PLoS One. 2020 May 6;15(5):e0232579. doi: 10.1371/journal.pone.0232579 (PMC7202631; doi:10.1371/journal.pone.0232579)
Supplement: Attachment — Submitted filename: Response_to_reviewers_09_02_2020.docx [file pone.0232579.s005.docx]

**February, 09, 2020**

**Re: Our revised manuscript PONE-D-19-23140**

**“The development of narrative skills in Turkish-speaking children: A complexity approach”**

Dear Reviewers,

We would like to thank you for your invaluable comments and relevant requests you made on our original manuscript. They were very helpful in improving the manuscript in terms of conceptual focus, methodological clarity, statistical analysis, and scope of interpretation.

We addressed all of comments individually, indicated as “Authors’ response:” in red right below each comment. The present document compiles all your comments and our responses so that you can retrieve your own ones as well as those of the other reviewers (if you wish). We hope we replied to your comments and made the relevant changes in the revised manuscript to your satisfaction.

We greatly appreciate the time you spent on our original manuscript and will spend again on reading our revised manuscript. Thank you very much.

With collegial regards,

the Authors

Reviewer #1:

Reviewer #1, comment 1: The theoretical motivation—the rationale-- for the study is underdeveloped. Theory of mind, executive function, and linguistic recursive ability are individually expected to predict a particular component or components of narrative complexity (pg. 8). However, the reason (i.e., theoretical motive) for testing all three predictors for all three aspects of narrative complexity in one study is not clear (other than no one has previously brought all six factors together in a study). For instance, at the top of page 8, recursive abilities are briefly discussed without a clear rationale that ties those abilities to one or more of the specific narrative complexity factors. Next, however, at the end of the Introduction (line 181), recursive ability is predicted to impact syntactic complexity in narrative. Later, the impact of recursive ability on plot complexity is the focus of analysis (pg. 19) leading to a somewhat post hoc explanation of the link between the two variables (pgs. 22 and 23).

Authors’ response: Thank you for pointing out these missing points in our manuscript. On page 3, we added our rationale more explicitly and emphasized that the time is ripe now to consider different domains of complexity together and to examine how the same set of predictors have the same or different effect on them is after these issues had been considered separately in the previous literature. Moreover, on Page 8 we discussed why recursive linguistic abilities were expected to be related to plot, evaluative and syntactic complexity.

Reviewer #1, comment 2: This is a minor point -- In the Introduction, it would be helpful to spell out more directly what ‘syntactic complexity’ means (i.e., are the authors talking about more than subordination?). For instance, reference is made (line 91) to developmental differences in syntactic complexity and it would be helpful to know the aspects they are referring to that bear on the present study.

Authors’ response: Thank you for reminding us of the unclear definition of syntactic complexity. As a measure, we only considered the ratio of complex C-units with subordinate clauses over the total number of C-units. This measure is concerned only with subordination. Thus, we modified the section on Syntactic complexity on Page 4-5 and reported only the findings of previous studies which defined syntactic complexity in terms of subordination, similar to our study. On Page 5, we also included our motivation for focusing on Turkish through describing the syntactic differences between Turkish and English (as one of the mostly studied languages).

Reviewer #1, comment 3: As the authors note, the age groups are imbalanced in number and by gender. There were age by gender interactions (S1 File). Because of the relatively low number of 4-year-old participants (n = 18) and the necessity of administering a separate theory of mind task for that age group, the 4-year-olds’ results are analyzed separately from the other age groups(Table 2). The low statistical power limits the analyses (and conclusions) for this age group (lines 398-401, pg. 19).

Authors’ response: Thank you for suggesting a power analysis. Using G*Power, we found that in general our regression analysis has a power of medium effect size (Effect size f2 = .13). We were careful not to over-interpret the results from the 4-year-olds.

Reviewer #1, comment 4: Inter-rater reliabilities are reported for the theory of mind tasks and the executive function task. For the sake of clarity, it would be helpful to include a sentence spelling out that the rater reliability for these tasks refers to agreement on correct and incorrect responses (assuming this is the case). For the other variables (which involve more than pointing to an answer or forced-choice responding), it is typical to report Cohen’s kappa in addition to interrater reliability. There are a number of suggestions in the literature for what constitutes an acceptable kappa.

Authors’ response: Thank you for pointing out the need for reporting Cohen’s Kappa regarding the results of the inter-rater reliability analyses. We added the scores whenever applicable. We also included a brief description of the consensus estimates method on Page 10.

Reviewer #1, comment 5: Please briefly clarify how the maximum score (19) is determined for the plot complexity coding (line 333, pg. 15).

Authors’ response: Thank you for pointing out the need for a detailed description of the plot complexity coding. We included a supplementary material (S1 Table) displaying the details of the coding and scoring. In this supplement, it is explained how the maximum score of 19 is attained.

Reviewer #1, comment 6: The evaluative complexity categories do not include perception (S1 Table). In the Shiro research that the authors cite, reference to perception was the most common evaluative device used by children. The perception category seems especially relevant to the present study given the authors’ interest in the impact of theory of mind on evaluative complexity.

Authors’ response: Thank you for pointing out that the perceptual mental state terms are not included as a category to code evaluative complexity. We excluded them because information regarding perceptual states (e.g. looking, hearing and smelling) is mostly present in the pictures of the book used for the elicitation of the narratives and the participants can refer to them without any deliberate evaluation. We stated the inclusion of perceptual states in the referential category in the section on the coding criteria for evaluative complexity.

Reviewer #1, comment 7: Please report the means and standard deviations by age group for the six major variables. It would also be helpful to see the zero order correlations among the variables. Without seeing those correlations, it is difficult to know whether there is a risk of multicollinearity in the regression analyses.

Authors’ response: Thank you for pointing out the need for the descriptive statistics on the variables. We now presented them in Table 2 on Page 19. We also presented the correlation coefficients in Table 3 on Page 21. We also checked the assumption of no multicollinearity and reported the relevant values on Page 20.

Reviewer #1, comment 8: The S1 file contains some information (such as the age by gender interactions) that could be easily integrated into the main text.

Authors’ response: Thank you for this suggestion. We reported the results of the revised analyses in the main text.

Reviewer #1, comment 9: Given the effect of gender on plot complexity (pg. 18), should gender be included as a predictor variable for the regression analysis for plot complexity (lines 402-403, pg. 19)? Also, if executive function was included in the regression analysis (line 402), it should be included in Table 3.

Authors’ response: Thank you for this suggestion improving our statistical analysis. Considering your and Reviewer 2’s suggestion, we reran our regression analyses including ‘gender’ as a predictor. We reported the new results accordingly.

Reviewer #1, comment 10: The Discussion could be more concise and focused on the specific contributions of the study.

Authors’ response: Thank you for this suggestion. We elaborated on our findings regarding the effect of gender on plot complexity, and the relationship between the domains of complexity and their predictors, thus highlighting the novelty of our study.

Reviewer #2:

Reviewer #2, comment 1: Introduction
I was missing some arguments on the research gap this study would like to address, and the novel contribution to prior research. Later in the paper, there are some good points on this, I would suggest to relocate them here to make the introduction more convincing and less descriptive.

Authors’ response: Thank you for pointing out the need to clearly specify the research gap and the novel contribution of our paper. Reviewer 1 raised a similar concern about the rationale of our study (1). We addressed this issue on Page 3. We explicitly emphasized that the time is ripe now to combine different domains of complexity and to examine how some social, cognitive and linguistic predictors have the same or different effect on each of them after these issues had been considered separately in previous literature.

Also, at first read it was not clear that the “domains of complexity” in narratives refers to narratives generated by the participants, not ready made narratives. This should be explicit upfront to help the reader’s understanding.

Authors’ response: Thank you for helping us avoid this ambiguity. We added “*children’s*” in the first sentence in the abstract which now reads as follows: “The present study examines the development of plot, evaluative and syntactic complexity in children’s narratives and its relationship with ToM, executive function and linguistic recursive ability.”

Reviewer #2, comment 2: Plot complexity
Based on the definition, it is not clear what makes this a complexity feature. In other words, what indicates higher and what indicates lower level of complexity. It only becomes clear towards the end of the paper.

Authors’ response: Thank you for suggesting to spell out earlier in the text in what sense plot complexity can be lower or higher. We added a clarifying sentence on page 3:

“Plot complexity is thus revealed in the extent to which the narrator realizes this hierarchical structure in her plot line.”

I am missing a concluding sentence, how does this all relate to the current study. Are there any predictions the authors could make?

Authors’ response: We are sorry that the relation of plot complexity with our study does not become clear already in this introductory section. However, rather than stating this connection here separately for plot complexity, we waited until the end of the introduction where we clarify this for all types of complexity. Still, we added a “summarizing” sentence that makes the present section more coherent: “The preschool and school years seem to be an important developmental period for increases in plot complexity.”

Reviewer #2, comment 3: Evaluative complexity
Same as above. Based on the definition, it is not clear what makes this a complexity feature. In other words, what indicates higher and what indicates lower level of complexity. It only becomes clear towards the end of the paper.

Same as above. I am missing a concluding sentence, how does this all relate to the current study. The paragraph indicates you are doing comparison of nationalities. The authors should conclude this section with a prediction, or at least with a link to the current study.

Authors’ response: Likewise, we explicitly stated what constitutes evaluative complexity already here. We added the following sentence in the section on evaluative complexity:

“Evaluative complexity results from this perspective taking. Both meta-cognitive and meta-linguistic abilities are required from children in order to conceive and syntactically express such evaluation.”

Reviewer #2, comment 4: Syntactic complexity
Line 88-87 implies that syntactic complexity is dependent of the other types of complexities. Theoretically it makes lots of sense, but it makes me wonder, if this affects the predictions. One would assume a high correlation among these variables.

Authors’ response: We agree with your assessment. This is particularly true for evaluative and syntactic complexity since evaluation requires complex syntax (subordination). In order to reveal the correlation among the three types of complexity, we added correlation tables, also including the predictor variables (Tables 3+4 for four-year-olds and older children, respectively). As you can see from Table 3, the correlation between evaluative and syntactic complexity is very high, *r*=.91*** in the 4-year-olds whereas it declines to *r*=.28** in the older children. We discussed this relationship on Page 32-33 considering the correspondence between the use of evaluative devices and the required syntactically complex structures.

Same as above. I am missing a concluding sentence, how does this all relate to the current study. The paragraph indicates you are doing comparison of nationalities. The authors should conclude this section with a prediction, or at least with a link to the current study.

Authors’ response: We agree that a conclusion pointing out the cross-linguistic aspect addressed in our study would strengthens the relevance of our paper. Therefore, in response to your request we added the following sentence at the end of the paragraph on syntactic complexity on Page 5.

“Considering these cross-linguistic differences and the lack of studies including syntactic complexity in Turkish narratives, our study aims to elucidate the development of syntactic complexity in Turkish-speaking children in relation to their narrative development.”

Reviewer #2, comment 5: Hypothesis
I am not convinced that all hypotheses are well grounded in the presented prior findings. I would suggest that the authors check and complete the argumentation whenever it is necessary.

Authors’ response: Thank you for pointing out the need for more clear argumentation for our hypotheses. On Page 8-9, we elaborated on the possible relationship between the linguistic recursive ability and the domains of the complexity.

There are many hypotheses, not always easy to follow, therefor it would be really helpful to see a visual overview of the conceptual model.

Authors’ response: Thank you for this suggestion. We constructed a visual overview of the expected predictors of each domain of complexity in S1 Figure.

Line 177-181: this section breaks the flow of the hypotheses, relocate it to the method section.

Authors’ response: Thank you for this suggestion. We relocated it on Page 13 under the section titled ‘Emotional Stroop Task’.

Reviewer #2, comment 6: Methods
Table 1, replace semi colons to dots when indicating mean age.

Authors’ response: Thank you for alerting us about the proper format how to report mean age. We changed it as you suggested.

Checking gender distribution among age groups: it is mentioned in the limitations in one sentence that gender was not distributed equally among age groups. I would like to see the exact calculation here upfront. Also, I believe that gender should be always included as a covariate into all statistical tests to control for this confound. The authors mention that the effect of gender was not significant (line 356), which is not supported by data, so we don’t know to what extent it is not significant. Nevertheless, gender has to be included because of the confound.

Authors’ response: Thank you for this suggestion improving our statistical analysis. Considering your and Reviewer 1’s suggestion, we rerun our analyses, included ‘gender’ in all ANOVAs and as a predictor in regression analyses. We reported the new results accordingly.

Also, I would like to see information on the distribution of schools (the location of recruitment) among the age groups. It looks like a multilevel data, i.e. it can be that students learning in better schools have better mental skills. The school itself can have an influence on the dependent variables. The authors should reflect on this theoretically but perhaps also statistically.

Authors’ response: Thank you for reminding us about the importance of schools from which the children were recruited as a possible confounding factor. The schools were public schools located in two districts of Istanbul (Findikzade and Beylikdüzü) with a population of middle socio-economic status. Due to the fact that the child participants were not evenly distributed between them when considered together with the other variables, we could not include ‘school’ as a variable in our statistical analyses. Since there were only three schools, a multilevel analysis (with school as a random factor) does not seem appropriate. To be able to control the effect of socio-economic status and other confounding variables such as the number of siblings, we asked the parents to fill a demographic information questionnaire. Unfortunately, only almost half of the parents filled this questionnaire, so we could not include the relevant details about our participants in our manuscript.

The authors should provide a power analysis for their study. Non-significant effects might be due to power issues.

Authors’ response: Thank you for this suggestion.We checked the power using G*Power and found that in general our regression analysis has a power of medium effect size, Effect size f2 = .13.

Reviewer #2, comment 7: Materials
Why is section 207-211 called Materials? It belongs to the coding procedure discussed later.

Authors’ response: Thank you for pointing this out. Due to the fact that we included the inter-rater reliability scores not only for the three domains of complexity in narrative, but also for ToM, executive function and linguistic recursivity tasks, we could not move this section on how the inter-rater reliability was calculated to where you suggested because mentioning it in the coding section would be too late.We hope this does not create any confusion in the reader.

Inter-rater reliability: throughout the paper, the authors are extremely vague about how inter-rater reliability was calculated. It seems they used simple percentage, which is not an adequate way to do it. I would suggest that authors recalculate IR reliability in terms of Krippendorf alpha or Cohen Kappa.

Authors’ response: Thank you for pointing out the need for reporting a more sound measure for inter-rater reliability. Considering your comment with the comment of Reviewer 1, we calculated Cohen’s Kappa and added the scores whenever applicable.

It is not clear at first how the sessions were recorded. Please, provide details.

Authors’ response: Thank you for reminding us of this point. We included the relevant details on Page 16.

Reviewer #2, comment 8: Second order ToM task
One or two examples would help the reader understand how this measure works.

Authors’ response: Thank you for reminding us about the need for the Second-order ToM task. We added the plot of one of the two stories and the questions asked about it to measure second-order ToM ability on Page 13.

Reviewer #2, comment 9: Comprehension of complement clauses task
When it comes to the result section it is difficult to trace back which construct was measured by which measure. I would suggest that to indicate the construct name in the sub-headings to make the readers’ task easier.

Authors’ response: Thank you for this suggestion. We added the name of the constructs on Page 14 (Executive Function Task, Linguistic recursivity Task).

Reviewer #2, comment 10: Syntactic complexity (line 345)
The authors say that the text was segmented into main and subordinate clauses. This can be tricky when verbal responses are transcribed and the transcriber puts there the punctuation that later serves as the basis for unit segmentation. Typically segmentation is also done by at least two people whose agreement is measured. I am very curious how the authors ensured that the segmentation is accurate (1. At the level of transcription 2. At the level of segmentation of the transcribed texts).

Authors’ response: Thank you for your concern regarding the difficulty in the segmentation of the narratives into main and subordinate clauses. Both the first author and the second rater transcribed the narratives. There was no difference between them in the segmentation of the narratives into C-units. The structure of the Turkish with the finite forms indicating mostly main clauses and non-finite forms indicating the subordinate clauses and the prosody of speech are helpful for them. Regarding the classification of the C-units into simple versus complex clauses, the suffixes inflected to the verbs or specific conjunctions used for the subordination were useful. We ran reliability analysis for this classification and reported its result on Page 17.

Reviewer #2, comment 11: Results
The authors should be consistent about how they define marginally and non-significant values. Line 406 p = .054 is defined as marginally significant; Line 419 p =.06 is defined as non-significant.

Authors’ response: Thank you for reminding us for this inconsistency. Because some of the hypotheses were about the relationships suggested for the first time in literature, we stayed conservative about their testing and tested them in two-tailed tests. Thus, even when results would have been significant under a one-tailed test, as it happened to be the case for the prediction of plot complexity by linguistic recursivity (*p* = .067). Consequently, we treated consistently *p* values over .05 as not significant.

Reviewer #2, comment 12: Discussion
Line 512: the lack of statistical power --- what do the authors mean by this here, does not seem to fit the argument.

Authors’ response: Thank you for warning us. We realized that we used “the lack of statistical power” incorrectly, thus we deleted it.
Line 536: Finnish is also an agglutinating language, it reads as if it was not and contrasted with Turkish.

Authors’ response: Thank you for pointing out this misunderstanding. We changed it accordingly.
Line 530-540: a few examples would help to follow the argumentation on the unique nature of Turkish language.

Authors’ response: Thank you for asking for examples. Syntactic complexity and the differences in the acquisition and the use of different syntactic structures in Turkish deserve more analysis and discussion which does not fit within the scope of this paper. We hope that the file including the syntactic categories coded for the present paper (S3 Table) and their examples will be helpful to you and the readers. Currently, we are working on a separate paper on syntactic complexity and discussing it in more detail in this paper.
Around line 590: the argumentation on the plot oriented style. I would suggest that the authors also reflect on their methods at least in two ways:
1. How the instruction was formulated, I would say that it was formulated in a way that triggered plot-oriented narratives as opposed to evaluation-rich narratives.

Authors’ response: Thank you for your concern about the effect of the instruction on the narratives told by our participants. The experimenter, the first author, explicitly told the participants that the narrative should include the emotions and thoughts of the story characters as well as the events. We added this detail in the Method section on Page 11. Moreover, we discussed he plot-oriented style of our participants despite this instruction on Page 31.

2. Adults had to comment on a children book, which largely decreases the ecological validity of the narrative they generated. They most probably will not evaluate children book stories and characters not because of their cultural background but because of the simplicity of the story. Perhaps there are other studies of the same kind with Turkish adults that could help to understand what was going on.

Authors’ response: Thank you for your concern about the ecological validity of the narrative task for our adult control group. However, concerning this issue we disagree with you. The story “Frog, where are you?” is by no means a simple children’s book. It combines, in an intricate way, three classical plots – a “lost-and-found” plot (the frog runs away from the boy and is found again at the end), a “friendship” plot (friendship between the boy and his dog) and a “romantic” plot (the frog finds a lady frog and founds a family). The romantic plot is not really well understood by most children. They mostly do not catch the twist at the end – that the boy takes one of the baby frogs back home and not the original frog – who is now a grown-up parent frog. Please also note that “Frog, where are you?” has been used with adults also in many studies before. For an overview see: <https://childes.talkbank.org/access/Frogs/>.

If you would like to “read” the book, please find it here:

<https://www.phil-fak.uni-duesseldorf.de/fileadmin/Redaktion/Institute/Allgemeine_Sprachwissenschaft/Frogstory-2_01.pdf>

Reviewer #2, comment 13: Conclusion
As I mentioned above, gender is a confounding variable, which should be controlled for which might change the outcomes and hence the discussion.

Authors’ response: Thank you for reminding us of this point. Reviewer 1 had the same concern. As indicated above, we now included gender in all our analyses.

Reviewer #3:

This is a brilliant paper, and I am looking forward to seeing this published so that I can refer to it in my own work. I found it very enlightening and clearly structured. There are a few concerns that I would like to share with the authors in the hope that they can use my feedback in their final submission.

Authors’ response: Thank you for your kind appreciation of our manuscript. We feel encouraged to improve it even more in the light of your and the other reviewers’ suggestions.

Reviewer #3, comment 1: I think it is important to pay attention to the possibility of intra-cultural differences (page 4, line 76) and where they might come from, either here or in the discussion. What would the parameters be of such differences? Exposure (quality/quantity) to narratives? Being read to as a child? Obviously narrative skills are essential, they seem to develop somehow, but… how? I very much hope the authors can share their knowledge about this. These parameters might also shed light on intercultural differences.

Authors’ response: Thank you for pointing out the importance of intra-cultural, inter-individual differences in children in our study. We fully agree with you that the factors you mention – quality and quantity of exposure – may influence children’s narrative productions. We had addressed this issue in the discussion already but stressed it with an additional phrase, on page 34.

Reviewer #3, comment 2: It is important to communicate with the readers what the rationale is for choosing Turkish speaking children. Is there any particular expectation here about their development in comparison to others? Are there theoretical reasons to focus on them? It might be just practical reasons of course! And I do not mean to say the choice requires justification.

Authors’ response: Thank you for pointing out the importance to clearly communicate the rationale of choosing Turkish children as participants. You are right in your assumptions: our choice is partly theoretically justified on typological grounds, as we explained in more detail in the revised manuscript and partly owing to the fact that the first author is a Turkish scientists and that both co-authors are working in Turkey, so we had privileged access to a Turkish sample. We believe both reasons are legitimate but stressed the first, primary one even more in our manuscript (e.g. on Page 3 and 5)

Reviewer #3, comment 3: - Page 10, line 213, I fully understand that it is necessary to work with the same book for all groups. But having 10-11 year olds and adults respond to a picture book about a frog might pose a serious problem. Personal experience suggest that kids of that age are less than willing to be involved in picture books. Could the authors reflect on that?

Authors’ response: Thank you for your concern. Reviewer 2 had a similar concern, see above. We are actually more optimistic on this issue. The story “Frog, where are you?” is not a simple children’s book. It combines three classical plots – a “lost-and-found” plot (the frog runs away from the boy and is found again at the end), a “friendship” plot (friendship between the boy and his dog) and a “romantic” plot (the frog finds a lady frog and founds a family). The romantic plot is not really well understood by most children. They mostly do not catch the twist at the end – that the boy takes one of the baby frogs back home and not the original frog – who is now a grown-up parent frog. Please also note that “Frog, where are you?” has been used with adults also in many studies before. For an overview see: <https://childes.talkbank.org/access/Frogs/>.

Reviewer #3, comment 4: Page 2, line 44. I am not sure whether it is necessary to use caps. Also later on page 7. There are also other keywords that are important in this article, and it is unclear why these words are privileged with the use of caps.

Authors’ response: Thank you for these concerns about the proper format. We revised it as you suggested.

Reviewer #3, comment 5: Page 8, line 177, maybe explain here briefly what an emotional Stroop Task is. Also, what “the rule” refers to might not be clear to all readers.

Authors’ response: Thank you for pointing out this ambiguity at this point in the text. The emotional Stroop Task is explained in the following paragraph. Therefore, we just added an explanatory clause in the critical sentence: “(the rule) how to respond to the stimuli...”

Reviewer #3, comment 6: Unclear to me what the numbers under M refer to in table 1. 4;4, should that not be 4.4? And M’s should be accompanied by SD’s.

Authors’ response: Thank you for alerting us about the proper format how to report mean age. In line with Reviewer 2’s suggestion, we substituted the previous notation “year;months” with the proper notation “year.months” in order to denote mean years.

Reviewer #3, comment 7: Page 9, line 196: “Most children” is a bit vague. Especially important considering the potential influence of background differences within a culture.

Authors’ response: Thank you for your concern about the effect of socio-economic status on our findings as a confounding variable. We recruited our child participants from two districts of Istanbul, namely Findikzade and Beylikdüzü. Both of them have a population of families of middle socioeconomic status. In addition to be able to control the effect of socio-economic status and other confounding variables such as the number of siblings, we asked the parents to fill a demographic information questionnaire. Unfortunately, only almost half of the parents filled this questionnaire, so we could not include the relevant details about our participants in our manuscript and control for the possible effect of these variables.

Reviewer #3, comment 8: Could the authors maybe provide a bit more information about the coding? One student did the coding? Where did the differences then occur? Between who and who? The student and the authors?
Authors’ response: Thank you for asking for more detail about the coding procedure. Reviewer 1 and 2 had similar concerns (see above). We provided more detailed information about the coding in the manuscript on Page 11.

Reviewer #3, comment 9: Do mention somewhere the potential threats of validity when cells are so small (table 1: 2 adult males, only 7 females in age group 4).
Authors’ response: You are right and we addressed the unequal gender distribution in the limitations, as you requested.

Reviewer #3, comment 10: Maybe I overlooked the explanation of why adult participants had to be excluded from the analyses. (p. 16, line 352).

Authors’ response: We are sorry that our decision not to include the adult group in the statistical analysis was not communicated sufficiently clear. We had stated the fact that adults were excluded from the statistical analysis and that their data was only being included in the descriptive statistics at the beginning of the results section – however, without any explicit reason. On Page 18, we now added an explanation, as follows:

“Data from adult participants were excluded from the statistical analyses in order not to distort the results of the children, but included in the figures or tables for only descriptive purposes so that the child participants’ performance can be compared to the performance of the adult participants.”

Reviewer #3, comment 11: Please check the quality of Figure 1. It was a bit hard to read when printed on paper.

Authors’ response: Thank you for your concern about the quality of Figure 1. Considering the suggestion of Reviewer 1 and Reviewer 2, we substituted it with Table 2 displaying the descriptive statistics in a more clear way.
